# Cysteine-Capped Hydrogels Incorporating Copper as Effective Antimicrobial Materials against Methicillin-Resistant *Staphylococcus aureus*

**DOI:** 10.3390/microorganisms8020149

**Published:** 2020-01-21

**Authors:** John Jackson Yang, Yung-Chi Huang, Tsung-Hsien Chuang, Deron Raymond Herr, Ming-Fa Hsieh, Chun-Jen Huang, Chun-Ming Huang

**Affiliations:** 1 Department of Life Sciences, National Central University, Taoyuan County 32001, Taiwan; johnjacksonyang@gmail.com (J.J.Y.); assassin89757@hotmail.com (Y.-C.H.); 2 Immunology Research Center, National Health Research Institutes (NHRI), Zhunan, Miaoli County 35053, Taiwan; thchuang@nhri.edu.tw; 3 Department of Pharmacology, National University of Singapore, Singapore 117543, Singapore; phcdrh@nus.edu.sg; 4 Department of Biomedical Engineering, Chung Yuan Christian University, Taoyuan City 32023, Taiwan; 5 Department of Biomedical Sciences and Engineering, National Central University, Taoyuan County 32001, Taiwan; cjhuang@ncu.edu.tw

**Keywords:** antimicrobial, copper, cysteine-capped hydrogels, *S. aureus*, skin wounds

## Abstract

Methicillin-resistant *Staphylococcus aureus* (*S. aureus*) (MRSA) has become an alarming threat to public health, and infected soft tissue. Antibiotics are commonly used to treat skin infection with MRSA, but the inappropriate use of antibiotics runs a considerable risk of generating resistant *S. aureus*. In this study, we created a cysteine-capped hydrogel able to absorb and release copper, an ion with the capability of suppressing the growth of USA300, a community-acquired MRSA. The results of analysis of Fourier transform infrared spectroscopy (FTIR) revealed the binding of copper to a cysteine-capped hydrogel. The topical application of a cysteine-capped hydrogel binding with copper on USA300-infected skin wounds in the dorsal skin of Institute of Cancer Research (ICR) mice significantly enhanced wound healing, hindered the growth of USA300, and reduced the production of pro-inflammatory macrophage inflammatory protein 2-alpha (MIP-2) cytokine. Our work demonstrates a newly designed hydrogel that conjugates a cysteine molecule for copper binding. The cysteine-capped hydrogel can potentially chelate various antimicrobial metals as a novel wound dressing.

## 1. Introduction

Pathogenic *Staphylococcus aureus* (*S. aureus*) can cause numerous human diseases [1]. This serious situation is predominantly due to antibiotic-resistant strains [2]. Methicillin-resistant *S. aureus* (MRSA) is one of the foremost causes of hospital- and community-associated infections. Resistance to the whole category of β-lactam antibiotics, like amoxicillin and methicillin, makes MRSA infections tough to treat [3]. The methicillin-sensitive (MSSA) and methicillin-resistant form (MRSA), an expedient infective pathogen agent responsible for most superficial skin infections, leads to enhanced morbidity, mortality, and extraordinary health care costs [4].

Current treatment choices for MRSA skin and soft-tissue infections embrace the utilization of antibiotics and the draining of abscesses. Clindamycin or cotrimoxazole remain the antibiotics of choice for less serious, non-multiresistant MRSA infections [5]. Vancomycin remains the foremost counseled antibiotic for MRSA infections. Telavancin, an additional recently developed derivative of vancomycin, may have similar clinical efficacy to vancomycin. Vancomycin or teicoplanin are employed for severe MRSA and multiresistant infections [5,6]. Some of the core issues that make antibiotic treatment difficult include the lack of selectivity, decreased vulnerability over time, and rapid antibiotic resistance. Furthermore, the virulence factor of MRSA, or its ability to cause illness, might be important if the bacteria gain entry into vulnerable body sites, like massive open wounds, invasive devices, or the bloodstream. In these conditions, the bacteria might cause opportunistic infections in vulnerable patients [7]. MRSA is one of the most common and recalcitrant pathogens compromising wound healing. Bamra reported that 75% of all patients with wounds that presented to their emergency department cultured positive for MRSA, demonstrating an area of high prevalence [8]. MRSA infections mostly occur in people who have been in medical centers or other health care settings, such as convalescent homes and dialysis centers. MRSA infections are typically related to invasive procedures or devices, such as surgeries, intravenous therapy, or artificial joints.

Skin is an important organ to regulate the body’s internal environment. When the skin is harmed or injured, the underlying tissue is uncovered and this significantly increases the chance of MRSA infections. Wound healing factors, such as fibroblast growth factor (FGF), vascular endothelial growth factor (VEGF), transforming growth factor-β (TGF-β), and epidermal growth factor (EGF), secreted from the fibroblasts, lymphocytes, neutrophils, macrophages, or platelets, can regulate the process of wound healing [9]. Hydrogels are hydrophilic three-dimensional (3D) networks of polymer chains broadly used in tissue engineering and drug delivery. Hydrogels were very desirable as tissue engineering scaffold and wound dressing candidates due to their biomimetic mechanical strengths, high water content, and the ability to mimic the interstitial tissue environment [10]. Several techniques have reportedly been able to improve the mechanical properties of zwitterionic hydrogels. These hydrogels include double network hydrogels, full-/semi-interpenetrating network hydrogels [11], and nanocomposite hydrogels [12]. Many such products are obtained from hydrocolloids, alginate, polyurethane, silicone, or nylon and may contain antiseptics such as iodine and silver. However, iodine and silver hydrogels, as dressings, are not suggested for chronic applications [13]. In a new approach to developing hydrogels, introduced by Haraguchi et al., exfoliated Laponite nanoclay (NC) platelets were dissolved in monomer solutions with an initiator and continued by polymerization [14]. NC hydrogels exhibit excellent mechanical, optical, and swelling properties, but they are not easily controllable due to batch-to-batch variations in preparations [14].

Azari et al., functionalized polyamide membranes with amino acids [15]. Amino acids are known to bind metal ions [16]. Amino acid zwitterions have been shown to chelate metal ions very well, with the charged amine and carboxylic acid acting as coordination ligands. These interactions have been used to make metal chelation gels. Metal chelation gels have also been prepared using the R group functionality of histidine [17]. The high affinity of histidine’s imidazole ring for metals is necessary for enzyme cofactors and protein folding [16]. Moreover, to these essential applications, adding amino acid functionality to polymers in a directed way can provide a means to tune metal-polymer interactions, and to add metal ion assembly properties to the resulting materials. In recent years, many studies have chosen natural amino acids as materials for making hydrogels, not only because amino acids are natural compounds but also because they have carboxyl groups (-COOH^−^) and amino groups (-NH_2_), which can be chemically conjugated to metal ions. The ions are detached or combined to form a negatively charged (-COOH^−^) and positively charged (-NH_3_^+^) as a natural amphoteric double ion. Of the common amino acids, cysteine (Cys), methionine (Met) and glutathione (GSH) can be chemically self-assembled with metal surfaces because of their thiol groups. Molecular membranes are valuable for bonding, wherein cysteine with high biocompatibility is a natural amino acid amphoteric double ion [18].

In the human body, copper is one of the essential elements. The lack of copper can lead to disturbances in growth and metabolism. Since copper in the body promotes iron absorption, insufficient copper intake can lead to anemia. Insufficient copper can also cause diseases such as neutropenia, osteoporosis, diarrhea, increased cholesterol, and thyroid disease [19]. Hydrogels with copper as antibacterial biomaterials have been reported [20,21,22]. However, the in vivo efficacy of most copper-integrated hydrogels has been validated. Mirastschijski et al., showed that the serum concentration of copper on preoperative day 0 and postoperative day 7 in acute wound patients was 17.8 µm and 19.0 µm [23]. Metal ions and metalloenzymes featured conspicuously during wound healing and experimental and clinical studies indicated that deficiencies in zinc, copper, calcium, iron, or magnesium were potential causes of irregular homeostatic mechanisms, and impaired wound healing [24,25]. Bacteria are briskly killed on copper surfaces, and copper ions released from the surface are said to play an important role in the killing process. In laboratory experiments, it was shown that many bacterial species, such as *Escherichia coli* O157, *S. aureus*, *Salmonella enterica*, *Campylobacter jejuni*, *Clostridium difficile*, *Listeria monocytogenes*, and *Mycobacterium tuberculosis*, are efficiently killed on copper or copper alloy surfaces [26]. Furthermore, copper is known to stimulate the maturation of collagen, thus it is crucial for improving the skin’s physical properties and thickness. In contrast to the microbes that developed antibiotic resistance in less than fifty years, microbes tolerant to copper are extremely rare, despite copper having been part of the world for millions of years. This lack of resistance to copper could also be explained by the capacity of copper to damage, in parallel, many critical factors in micro-organisms [22].

In this study, we synthesized cysteine-capped hydrogels as wound dressings with antibacterial ability against USA300, a community-acquired MRSA, based on their ability to absorb and release cooper ions [27]. The cysteine-capped hydrogels effectively suppressed the growth of USA300 *in vivo*.

## 2. Materials and Methods

### 2.1. Ethics Statement

This study was carried out in strict accordance with an approved Institutional Animal Care and Use Committee (IACUC) protocol (NCU-106-016, 19 December, 2017) at National Central University (NCU), Taiwan.

### 2.2. Synthesis of Cysteine Methacrylate (CysMA)

L-cysteine amino acid powder (ACROS organics, Geel, Belgium) (15.13 g) was dissolved in deionized water (100 mL). Subsequently, 29.43 g 3-(acryloyloxy)-2-hydroxypropyl methacrylate (MA) (TCI, Tokyo, Japan) and 20 μL dimethylphenyl phosphine (Alfa Aesar, Haverhill, Massachusetts, USA) were added. The aqueous mixture was stirred for 2 h at room temperature. It was then washed twice with 50 mL ethyl acetate (Sigma, St. Louis, MO, USA) and 50 mL dichloromethane (J.T Baker, New Jersey, USA) extracted in a separatory funnel. The final product was isolated by freeze-drying [28].

### 2.3. Preparation of CysMA/Copper Chloride Hydrogel and 3-(acryloyloxy)-2-hydroxypropyl Methacrylate/Copper Chloride Hydrogel

The protocol for the preparation of the hydrogel was according to a previous publication [12]. First, 20 μL of homogenized nanoclay (Rockwood Ltd., Newton Abbot, UK) (78.6 mg) in deionized water (1 mL) was added to a homogenized solution of 1 mL of 0.5 M CysMA or 1 mL of 0.5 M MA, followed by 20 μL poly (ethylene glycol) methacrylate (Sigma), 5 mg potassium peroxydisulfate (KPS) (ACROS) and 1 μL tetramethylethylenediamine (TEMED) (ACROS). The mixed solution was then added to the mold, until the end of the reaction. After the reaction, the hydrogels were removed and immersed in a phosphate buffer solution (PBS) for 12 h to remove non-reacted substances. The cleaned hydrogels were then cut into a 1 cm diameter disc shape before being soaked in 1 mL copper chloride solution (1 M) (Sigma) for 24 h. The CysMA hydrogels were then removed from the copper chloride solution and immersed again in a PBS overnight or used directly for the mouse experiment [29].

### 2.4. Fourier Transform Infrared Spectroscopy (FTIR) Analysis

After the hydrogels were drained in a vacuum and pulverized, they were mixed evenly with potassium bromide (KBr) (Sigma) at a ratio of 1:99 and compacted into a thin disk. The measurement of Fourier transform infrared spectroscopy (FTIR) was determined by Jasco FT/IR-410, measuring wavelengths from 400 to 4000 cm^−1^ at room temperature.

### 2.5. Copper Ion Absorption and Release 

The copper ion absorption and release were measured using the calibration curve of optical density (OD) value (610 nm) [30]. The hydrogel containing copper ions was soaked three times in 1 mL PBS to wash away free surface copper ions. The hydrogels were removed and placed in a new 1 mL PBS, copper solution were measured. After 24 h, the hydrogels were removed, and the solution was used for OD value detection.

### 2.6. The Antibacterial Activity of Hydrogel Containing Copper Ions

The antibacterial effect of hydrogels containing copper ions was examined on agar plates inoculated with USA300 [27] or MRSA252 [31]—a community- or hospital-acquired MRSA, respectively—using inhibition zone tests. Bacteria were cultured on 3% tryptic soy broth (TSB) (Sigma), at 37 °C. The bacterial solution was diluted to 10^6^ colony-forming unit (CFU) with fresh TSB. The TSB agar plate was prepared and 10 mL of USA300 solution containing 10^6^ CFU was spread evenly before the hydrogel was placed on a TSB agar plate. The cultures were incubated at 37 °C for 24 h to assess the inhibition zone for each hydrogel [32].

### 2.7. Minimum Bactericidal Concentration (MBC) Assays

*S. aureus* (10^6^ CFU/mL) was incubated in a copper chloride solution at various concentrations (10 μM - 1 M) in media on a 96-well microtiter plate (100 μL per well) for 24 h. Bacteria was incubated with PBS alone as a control. After incubation, the bacteria were diluted at 1:10^1^ to 1:10^5^ with PBS. The number of bacteria was quantified by spotting the dilution (5 μL) on an agar plate supplemented with media for the counting of CFUs.

### 2.8. Wound Size Infection on Mouse Animal Model

The Institute of Cancer Research (ICR) mice (8–12 month-old females; National Laboratory Animal Center, Taiwan) were anesthetized by isoflurane. Following shaving with electrical clippers, a 1 cm wound was made on the dorsal skin using sterilized surgical scissors (Fisher Scientific, Loughborough, UK) [27,33,34]. Following skin wounding, we applied 10 µl USA300 (10^6^ CFU) in PBS. The wound was covered by the hydrogel and fixed with breathable tape. To observe the wound size, a transparent parafilm was placed over the wounded skin and the area was marked by outlining the area of the wound. The lesion size (%) was measured daily and calculated with ImageJ software 1.50b (National Institutes of Health (NIH), Bethesda, MD, USA) [34].

### 2.9. Bacterial Counting

Three days after infection the mice were sacrificed. The excised skin was homogenized in 200 µl of sterile PBS with a tissue grinder. Bacterial CFUs in the skin were enumerated by plating serial dilutions (1:10^1^–1:10^5^) of the homogenate on a TSB agar plate. The plate was incubated overnight at 37 °C to count colonies. The bacterial numbers (CFUs/mL) of excised skin were calculated. The pro-inflammatory macrophage inflammatory protein 2 (MIP-2) cytokine was determined by sandwich enzyme-linked immunosorbent assay (ELISA) using a Quantikine mouse MIP-2 set (R&D Systems, Minneapolis, MN).

### 2.10. Statistical Analysis

To determine significance of the groups, comparisons were made using the two-tailed *t*-test. For in vivo experiments, five mice per group per experiment were used. The data represent the mean ± standard deviation (SD) from three independent experiments. For all statistical tests, the *p*-values of <0.05 (*), <0.01 (**), and <0.001 (***) were accepted as statistically significant.

## 3. Results and Discussion

### 3.1. Preparation of CysMA Monomer

In recent years, related research has been conducted to investigate the preparation of functionalized soluble amino acid polymers into hydrogels and membranes. Azari et al., modified commercial membranes using zwitterionic amino acid L-cysteine in a two-step process, involving the activation of the membrane with an epoxy compound [allyl glycidyl ether (AGE)], followed by the thiolene reaction to graft L-cysteine on the AGE activated membrane. Subsequently, Alswieleh et al., used functionalized cysteine as a bridge to form a double ion brush [28] with decent biocompatibility and stability.

**Figure 1:**
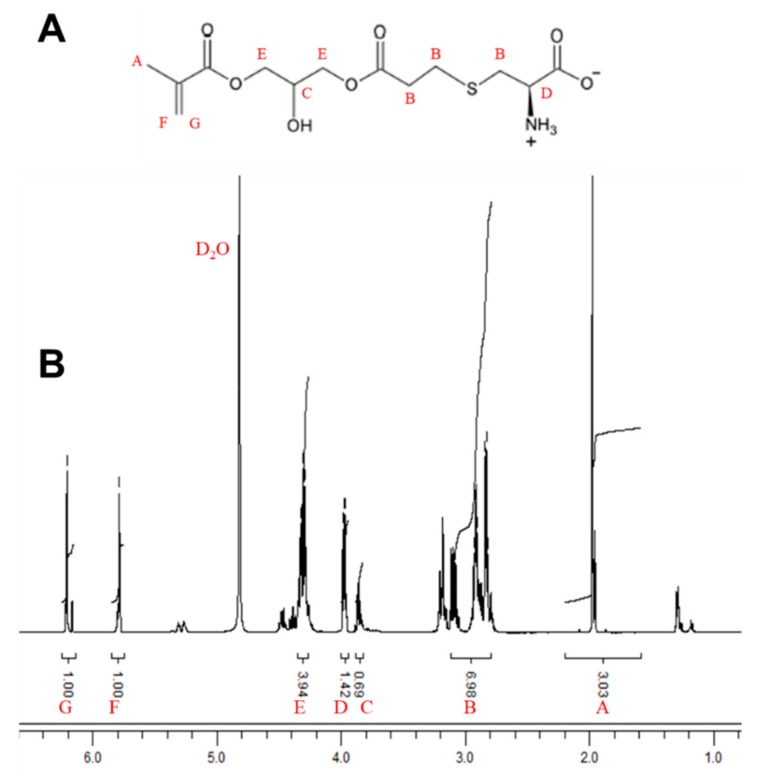
**^1^**H-NMR analysis of CysMA (500 MHz). The CysMA monomer was synthesized using a selective Thia–Michael addition in an aqueous solution at 20 °C. (**A**) CysMA structure. (**B**) The CysMA **^1^**H-NMR spectra. (400.13 MHz, D_2_O, 298 K) δ (ppm): [A] 1.89 (s, 3H, -CH_3_); [B] 2.68–3.17 (m, 6H, -S-CH_2_-CH_2_-COO-, -S-CH_2_-CH(COO_-_)NH_3_^+^); [C] 3.79 (m, IH, CHOH); [D] 3.90 (m, 1H, -CH(COO-) NH_3_^+^); [E] 4.20–4.30 (m, 4H, -CH_2_-CHOH-CH_2_-); [F] 5.70 (s, 1H, vinyl); and [G] 6.13 (s 1H, vinyl).

In this study, a CysMA monomer was synthesized using a selective Thia–Michael addition in an aqueous solution at 20 °C. L-cysteine, a natural amino acid, was reacted with 3-(acryloyloxy)-2-hydroxypropyl methacrylate. Dimethylphenyl phosphine (DMPP), an affinity reagent, catalyzed the reaction to form a CysMA monomer with an overall yield of 94% in 2 h. The chemical structure of the purified monomer was confirmed by ^1^H-NMR spectral analysis (Figure 1). The chemical shifts δ (ppm) of the ^1^H- nuclear magnetic resonance (NMR) spectrum of CysMA monomer (400.13 MHz, D_2_O, 298 K) are as follows: 1.89 (s, 3H, -CH_3_); 2.68–3.17 (m, 6H, -S-CH_2_-CH_2_-COO-, -S-CH_2_-CH(COO_-_)NH_3_^+^); 3.79 (m, 1H, CHOH); 3.90 (m, 1H, -CH(COO-)NH_3_^+^), 4.20–4.30 (m, 4H, -CH_2_-CHOH-CH_2_-); and 5.70 (s, 1H, vinyl), 6.13 (s 1H, vinyl). Their peak assignments are also indicated in Figure 1. In previous studies, it was confirmed that this method successfully synthesized cysteine methacrylate. The synthesis process was carried out in an aqueous solution without the need to protect specific functional groups. Compared to other zwitterionic polymer brush systems, the cost of, for example, poly(2-(methacryloyloxy)ethylphosphorylcholine) (PMPC) is significantly less [28].

### 3.2. Preparation of CysMA and MA Hydrogel and the Hydrogel Appearance

**Figure 2:**
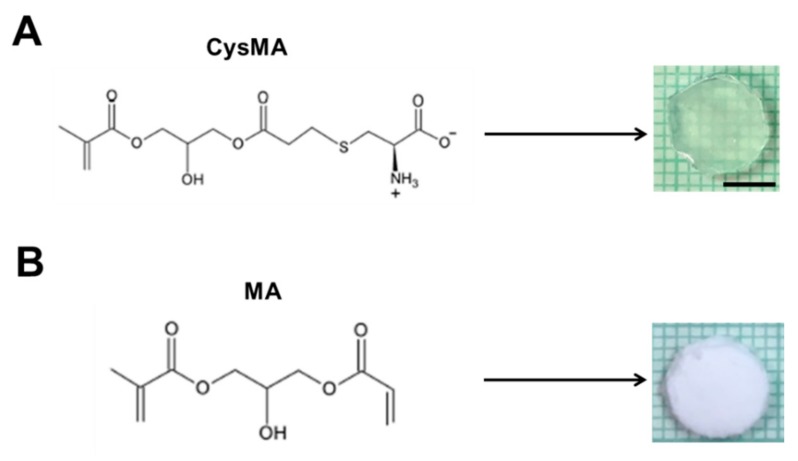
Preparation of hydrogels; 20 μL of homogenized nanoclay (78.6 mg) in deionized water (1 mL) were added to homogenized (**A**) CysMA or (**B**) MA 1 mL (0.5 M), followed by Poly(ethylene glycol) methacrylate (20 μL), KPS (5 mg) and TEMED (1 μL). The mixed solution was then added to the mold, waiting for the end of the reaction. Bar = 0.5 cm.

The preparation of the hydrogel is described in Section 2.3. This was used to polymerize a functionalized CysMA to form a polymer chain, and the polymer chain was connected in a series to form a network structure of hydrogel. In an early step, the surface amine and carboxylic acid groups were reacted with allylglycidyl ether, and were subsequently functionalized with cysteine’s thiol group using thiol–ene chemistry. Zhi et al., modified polyL-dopamine, which was coated onto a poly (ethylene terephthalate) (PET) sheet with lysine using Michael addition and yielding a zwitterionic surface that showed increased haemocompatibility relative to PET [35]. These three instances resulted in the successful functionalization of polymer surfaces with amino acid moieties. However, this modification was extensive and it took a long time for the surface treatment to enable the attachment of amino acids onto the surface. Very recently, Xu et al., synthesized hydrogels containing glycidyl methacrylate (GMA) on the surface of a hydrogel with the amine group of unprotected amino acids [36]. The interesting aspect of this work is that, by varying the amount of GMA in the hydrogel, they were able to functionalize this material without additional surface treatments and with nine unprotected amino acids with varied amounts of incorporation. The amino acids they incorporated into their compounds had their side chain groups preserved, enabling them to influence the adsorptive properties in a way similar to that in natural peptide systems. It has been shown that amino acids with zwitterions can be chelated with metal ions to form hydrogel (lysine) [17]. The CysMA monomer in this study was mainly reacted with cysteine and 3-(acryloyloxy)-2-hydroxypropyl methacrylate. The mixed solution of nanoclay, poly (ethylene glycol) methacrylate, KPS and TEMED, with the CysMA monomer or with only MA, was molded and cleaned in PBS for 12 h to remove unreacted material and surface free impurities. A mold cut in the shape of a disk, with a diameter of 0.8 cm, a height of 0.1 cm, and the surface of the hydrogel, was observed to be translucent for CysMA hydrogel (Figure 2A). On the other hand, the appearance of MA hydrogel was observed to be white opaque (Figure 2B).

### 3.3. Absorption and Release Ability of CysMA and MA Hydrogels

Hydrogels are hydrophilic macromolecular networks that are made by the chemical or physical crosslinking of soluble polymers. The unique characteristics of hydrogels, such as high-sensitivity to physiological environments, hydrophilic nature, soft tissue-like water content, and adequate flexibility, make them perfect candidates for biomedical applications. Hydrogels can swell and condense the solution in a reversible direction, showing specific stimuli-responsiveness, e.g., temperature, pH, and ionic strength. The smart physiological reaction of hydrogels toward changes in physiological factors promotes their utility for several biomedical applications [37].

**Figure 3:**
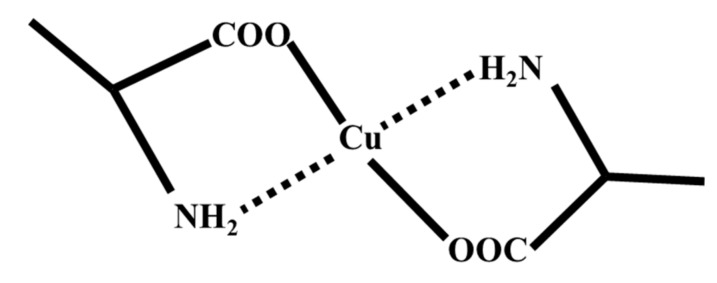
Copper ion and amino acid binding with chelate.

The redox chemistry of Cu^2+/+^ makes it essential for biological processes. However, it is potentially dangerous if not handled correctly by the cell, and becomes available for Fenton-like chemistry to produce reactive oxygen species (ROS). Typically, under the oxidizing extracellular environment, copper exists as Cu^2+^, but within the reducing conditions inside the cell, it exists in the reduced Cu^+^ oxidation state. Its soft character makes Cu^+^ distinctive among the biological metal ions. Therefore, it has the potential to be a selected supported ligand donor group. When the formation medium is basic, and there is no hydrogen interference, the carboxylic groups and amino groups on both sides of the polymer chain can bond with the metal atoms to form a chelate (Figure 3.) [38].

**Figure 4:**
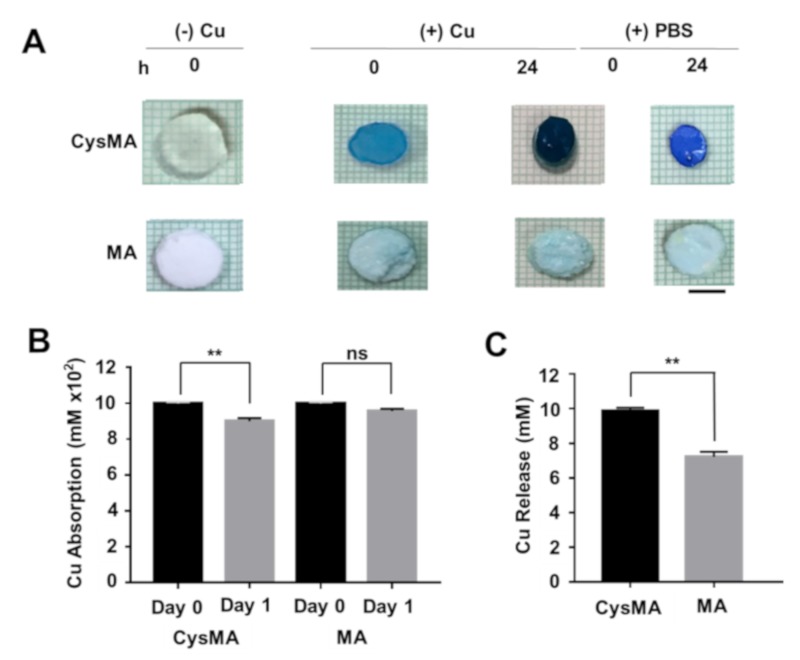
Copper ion absorption and release rate. (**A**) The hydrogels containing copper ions were soaked three times in 1 mL PBS to wash away free surface copper ions. The hydrogels were removed and placed in a new 1 mL PBS, and copper solution were measured (**B**). After 24 h, the hydrogels were removed and (**C**) the solution was measured using the calibration curve of OD value (620 nm). Bars = 0.5 cm. ** *p* < 0.01 (two-tailed t-tests). Data are the mean ± SD of three separate experiments. ns = non-significant.

To investigate the absorption and release ability of hydrogels, CysMA hydrogel and MA hydrogel were immersed in an aqueous solution of copper chloride (CuCl_2_, 1 M) for 2 h. Then, the hydrogels were taken out and immersed in PBS for 12 h to wash away the surface-free copper ions. The surface of the CysMA hydrogel was light blue and transparent, which was supposed to have the ability to absorb copper ions. Absorption of the copper ions increased along with increasing immersion time, and the surface color turned dark blue in the 24 h. This was followed by placing CysMA hydrogel in fresh PBS for 24 h, the CysMA hydrogel surface color changed from dark blue to light blue. There was no significant change in the surface color as the absorption time increased. After 24 h of absorption, the MA hydrogel was placed in fresh PBS for 24 h, and the surface color of the MA hydrogel was still white opaque (Figure 4A).

In this study, ultraviolet-visible (UV-Vis) spectroscopy was used for the calibration curve, absorption, and release of copper chloride [30]. The prepared CysMA and MA hydrogels were placed in a copper chloride solution (1 M), the hydrogels were taken out at a specific time, and the OD value of the remaining solution was detected by UV-Vis. Figure 4B showed that on day 0, the concentration of the CysMA hydrogel was about 1 M. After one day, the concentration decreased to 0.9 M, not significantly different for an MA hydrogel. Released copper ions were detected at 10 mM and 6.5 mM by measuring the PBS solution from immersed CysMA and MA hydrogels in sequence (Figure 4C). This result speculated that CysMA hydrogel has the ability to release, in addition to its absorptive capacity, but not for MA hydrogels. Due to leaching imperfections when removing the hydrogel from the copper solution to the new solvent, shown in Figure 4C, there is still copper detected at the MA hydrogel solution. It is possible that three repetitions of washing using PBS was not enough to remove the copper attached to the outer surface of the hydrogel. For further research, leaching in a competent solution is needed to wash unspecific binding copper to the surface of hydrogels.

### 3.4. FTIR Analysis

**Figure 5:**
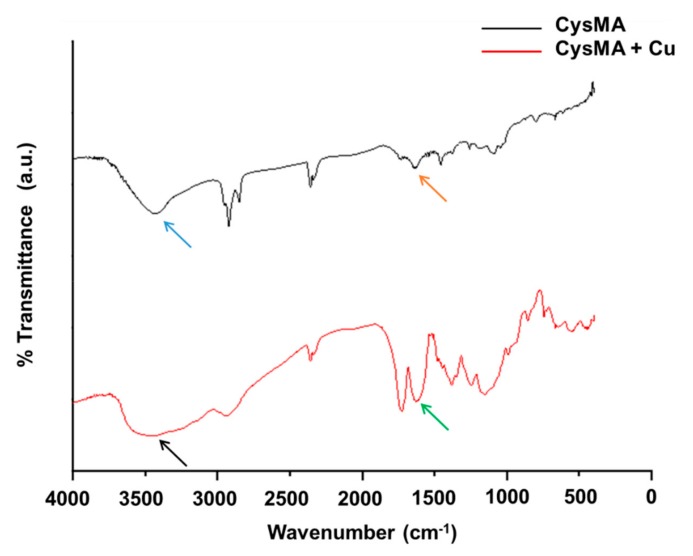
FTIR analysis. Two functional groups of hydrogels; COO- shifted from 1635 cm^−1^ (orange arrow) to 1626 cm^−1^ (green arrow), and that of NH_3_^+^, located at 3300–3500 (blue and black arrow), showed that hydrogels were chelated with copper ions; a.u. = arbitrary unit.

In order to investigate whether CysMA and metal copper ions were chelated, FTIR was used to analyze the change of characteristic peaks of CysMA with and without the addition of copper ions. In this study, the peak of the functional groups for the carboxylic group (COO-) was located at 1635 cm^−1^, while for the ammonium group (NH_3+_), it was located at around 3300 cm^−1^ (Figure 5). However, the intense peak of hydroxyl OH (3500–3560) of water contained in the hydrogel overlapped with that of the ammonium group (3300 cm^−1^). As such, the peak shift in the carboxylic group was used to determine the chelation of the hydrogel with copper ions, in this study. It has been shown that the divalent copper ion could chelate with the two functional groups (NH_2_ and COOH) of cysteine [39]. In addition, FTIR peaks of chitosan (CONH_2_) shifted from 1657 cm^−1^ to 1631 cm^−1^ when chitosan formed a complex with copper ions [40]. In this study, the functional groups on the hydrogel underwent a chelation reaction with the copper ion, resulting in the shift of the COOH peak from 1635 cm^−1^ to 1626 cm^−1^.

### 3.5. Antibacterial Test

The mechanism of copper ion sterilization was mainly due to the redox reaction of copper and copper catalyzing the production of lipid peroxidation, which in turn destroyed the biofilm [41]. Therefore, copper–oxygen complexes, which are directly involved in lipid peroxidation, can destroy many of the membrane-dependent active functions, such as transport protein activity, phagocytosis [42], and ion permeability. In another study [41], it was found that the bactericidal ability of copper is in different bacterial cell membranes, such as *Streptococcus lactis*, *E. coli*, and *P. aeruginosa*. The loss of metabolic function is the death of bacteria.

**Figure 6:**
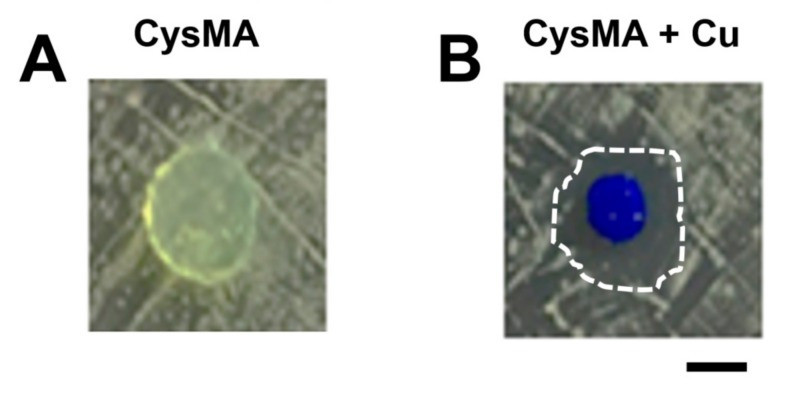
Antibacterial activity of copper released from hydrogels. The antibacterial activities of hydrogels without (**A**) or with (**B**) copper ions were examined on agar plates inoculated with MRSA, USA300, using inhibition zone (dashed circle) tests. Bar = 0.5 cm.

In this study, two hydrogels were prepared for the inhibition zone experiment. The CysMA hydrogel was immersed in a copper chloride (CuCl_2_) solution with 1 M concentration, or in PBS as a control group. Each group of hydrogels was placed on solid media containing USA300 (Figure 6) or MRSA252 (Figure S1) bacteria (10^8^ CFU, 10 μL) and co-cultured for 24 h. The results showed that in the CysMA hydrogel with copper ions there was a bacteria-free circle around the hydrogel, and this area was called an inhibition zone.

**Figure 7:**
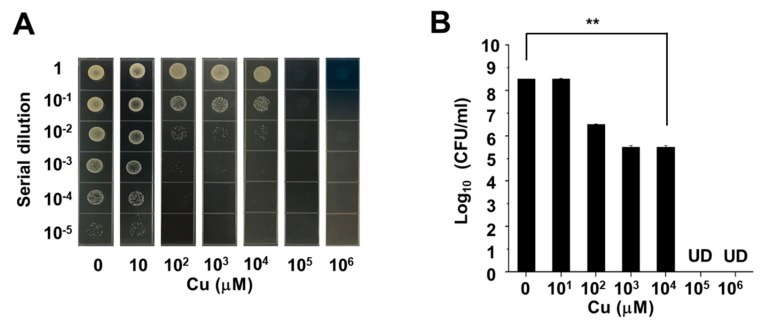
MBC assay of copper. Six different concentrations of copper chloride solution (10 μM, 10^2^ μM, 10^3^ μM, 10^4^ μM, 10^5^ μM, 10^6^ μM). The mixtures of the bacterial suspension and the copper chloride solution were enumerated by plating serial dilutions (**A**). The number (log_10_ CFU/mL) of USA300 were quantified (**B**). ** *p* < 0.01 (two-tailed t-tests). Data are the mean ± SD of three separate experiments.

In the other control group, the inhibition zone did not appear. Therefore, it was found that CysMA hydrogel immersed in a copper ion concentration of 1 M released copper ions after 24 h. This content had an antibacterial effect [22] in the inhibition zone test in an in vitro model widely used for the evaluation of the antibacterial activities of biomaterials [43,44]. In order to investigate the antibacterial ability of copper chloride for USA300, we carried out a minimum bactericidal concentration (MBC) assay. Six different concentrations of copper chloride solution (10, 10^2^, 10^3^, 10^4^, 10^5^, and 10^6^ μM) were prepared in this experiment, as well as a control group of PBS. The results showed that copper chloride achieved complete sterilization at a concentration of 10^5^ μM (Figure 7). Future work will use an electron microscope to determine the structural morphology of CysMA hydrogels and their interaction with MRSA to determine the minimum inhibitory concentration (MIC) of copper iron and CysMA hydrogel with copper ions, using the method of the Clinical and Laboratory Standards Institute (CLSI) or the European Committee for Antimicrobial Susceptibility Testing (EUCAST) [45,46,47].

### 3.6. Wound Infection Mouse Model

**Figure 8:**
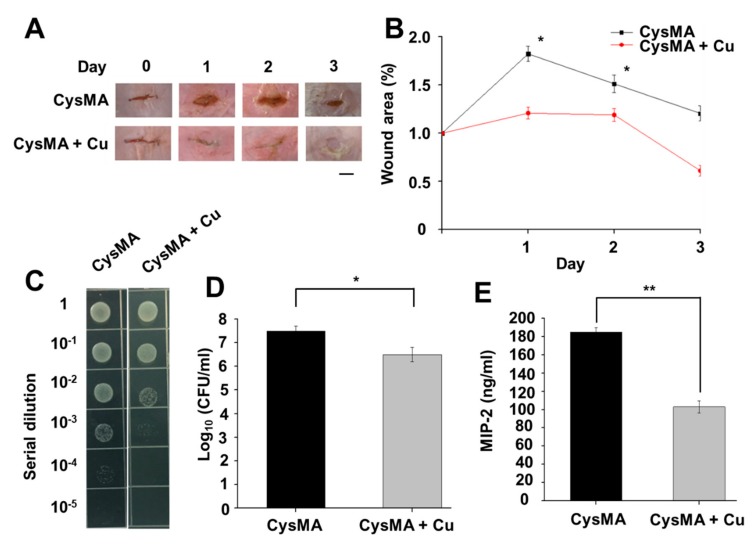
Mouse models of wound infections. A 1 cm wound infected with USA300 was created on the dorsal skin of an ICR mouse before the wound was covered by the hydrogels and fixed with breathable tape. Wound size was observed (**A**) and enumerated (**B**) daily. Bacterial CFUs in the skin wounds were enumerated by plating serial dilutions of the homogenate (**C**). The number (log_10_ CFU/mL) of USA300 (**D**) and the level of pro-inflammatory MIP-2 cytokine (**E**) were quantified. * *p* < 0.05, ** *p* < 0.01 (two-tailed t-tests). Data are the mean ± SD of three separate experiments.

In order to establish a wound infection model, we selected ICR mice for animal experiments and prepared two different sets of CysMA hydrogels. According to the results of the antibacterial experiment, CysMA hydrogel immersed in a copper ion concentration of 1 M, and a control group, were selected. The control group was a CysMA hydrogel soaked in PBS solution, and the bacteria were selected from USA300 (10^8^ CFU). The observed and enumerated wound are shown in Figure 8A, B. We found that the group of hydrogels containing no copper ions had marked redness and swelling after one day, which is a typical inflammatory phenomenon. In the group of hydrogel containing copper ions, there was no obvious redness or swelling in the wound on the second day, and the wound was slightly smaller than on the first day. After two days of observation, there was still inflammation in the group using the hydrogel containing no copper ions, and some tissue fluid was observed to leak out. In the group of hydrogels containing copper ions, the wound had begun to heal and no subcutaneous tissue could be seen. After three days of observation, in the hydrogel group containing no copper ions the inflammation was much slower than the two previous days and there was still tissue fluid. However, the wound had begun to heal and the wound size was smaller than the two previous days. In the hydrogel group containing copper ions the wound was almost invisible but a scar had formed in the skin tissue. When bacteria invade the subcutaneous tissue, they release toxins in the tissue and grow. Such a process can cause inflammation of the wound tissue and interfere with the rate of wound healing. In the area measurement experiment, CysMA hydrogel immersed in a copper ion concentration of 1 M was found to significantly contribute to wound healing in mouse experiments, and no typical inflammatory phenomenon occurred. This phenomenon was presumed to be due to the antibacterial effect of copper ions, which reduced the bacterial content in the wound tissue, slowed down the inflammatory response, and accelerated the rate of wound healing. Furthermore, results from terminal deoxynucleotidyl transferase dUTP nick end labeling (TUNEL) assays demonstrated that both CysMA hydrogels with or without copper ions had no cytotoxicities when they were topically applied to the dorsal skin of mice for 24 h (Figure S2). Borkow et al., hypothesized that the release of Cu from the wound dressing directly into the wound site would not only reduce the risk of wound contamination but, more importantly, would directly stimulate faster wound repair by inducing angiogenesis and skin regeneration [48]

To determine if the CysMA hydrogel with copper ions promoted antibacterial activity, skin wounds in mice were inoculated with USA300 bacteria. On the third day of the wound area experiment, the wounds were homogenized to measure the intensity of USA300 colonization. The results showed that the USA300 counts of CysMA hydrogel with and without copper ions were 3.6 × 10^5^ CFU/mL and 4.1 × 10^6^ CFU/mL, respectively (Figure 8C,D). It was confirmed, by the mouse experiments, that CysMA hydrogel containing copper ions has an antibacterial effect. Regarding the antibacterial ability of CysMA hydrogel, preliminary results were obtained and indicated that CysMA hydrogel with copper ions had antibacterial ability. It is known the binding of MRSA to Toll-like receptor (TLR)-2 provokes the secretion of pro-inflammatory cytokine of human IL-8, a counterpart of mouse MIP-2. As shown in Figure 8E, the level of MIP-2 in skin wounds was observed 3 days after USA300 inoculation. The CysMA hydrogel soaked in copper chloride solution demonstrated 55% less compared to CysMA hydrogel without copper ions.

In summary, we have successfully synthesized CysMA hydrogels containing copper ions. The cysteine-capped hydrogel can absorb and release copper ions, which have the ability to suppress the growth of MRSA. The topical application of CysMA hydrogels containing copper ions onto USA300-infected skin wounds in the dorsal skin of ICR mice significantly enhanced the wound healing, hindered the growth of USA300, and reduced the production of pro-inflammatory MIP-2 cytokine, demonstrating the in vivo efficacy of CysMA hydrogels with copper against USA300.

## Supplementary Materials

The following are available online at https://www.mdpi.com/2076-2607/8/2/149/s1, Figure S1. Anti-MRSA activity of copper release from hydrogels.; Figure S2. No significant cytotoxicities of hydrogels.
