# Supplementary material for: Cysteine-Capped Hydrogels Incorporating Copper as Effective Antimicrobial Materials against Methicillin-Resistant Staphylococcus aureus
Source: Microorganisms. 2020 Jan 21;8(2):149. doi: 10.3390/microorganisms8020149 (PMC7074715; doi:10.3390/microorganisms8020149)
Supplement: Supplementary file 1 [file microorganisms-08-00149-s001.pdf]

microorganisms

Supplementary Material

Cysteine-Capped Hydrogels Incorporating Copper as Effective Antimicrobial Materials against Methicillin-Resistant *Staphylococcus aureus*

John Jackson Yang [1], Yung-Chi Huang [1], Tsung-Hsien Chuang [2], Deron Raymond Herr [3], Ming-Fa Hsieh [4], Chun-Jen Huang [5], Chun-Ming Huang [5,*]

[1] Department of Life Sciences, National Central University, Taoyuan County 32001, Taiwan; johnjacksonyang@gmail.com (J.J.Y.); assassin89757@hotmail.com(Y.-C.H.)
[2] Immunology Research Center, National Health Research Institutes (NHRI), Zhunan, Miaoli County 35053, Taiwan; thchuang@nhri.edu.tw
[3] Department of Pharmacology, National University of Singapore, Singapore 117543; phcdrh@nus.edu.sg
[4] Department of Biomedical Engineering, Chung Yuan Christian University, Taoyuan City 32023, Taiwan; mfhsieh@cycu.edu.tw
[5] Department of Biomedical Sciences and Engineering, National Central University, Taoyuan County 32001, Taiwan; cjhuang@ncu.edu.tw
[*] Correspondence: chunming@ncu.edu.tw; Tel: +886-3-422-7151; Fax: +886-3-425-3427

1. Materials and Methods

1.1 TUNEL Assay.

To examine the cytotoxicities of hydrogels, dorsal skin of ICR mice was topically applied with a hydrogel with or without cooper for 24 h. Skin was excised, immersed and fixed in 10% formalin. Death cells in a 3 μm thick skin section was examined using a TUNEL assay kit (R&D systems) [1]. Briefly, biotinylated nucleotide was incorporated at the 3′-OH DNA ends of the fragmented DNA in dead cells. Horseradish-peroxidase-labeled streptavidin was bound to biotinylated nucleotides, which were detected using diaminobenzidine as a substrate to produce a dark brown reaction. To quantify the dead cells, a total of at least 3 random visual fields (150 μm x 150 μm) in a skin section was counted to quantify the dead cells.

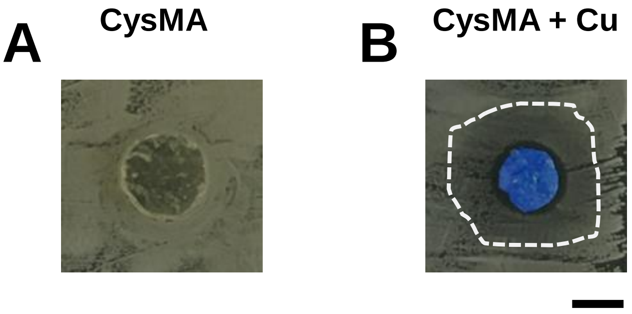

Figure S1. Anti-MRSA activity of copper release from hydrogels. The inhibition zone (dashed circle) tests were used to detect the antibacterial activities of hydrogels without (A, CysMA) or with (B, CysMA + Cu) copper ions on agar plates spread with MRSA252. Bar = 0.5 cm.

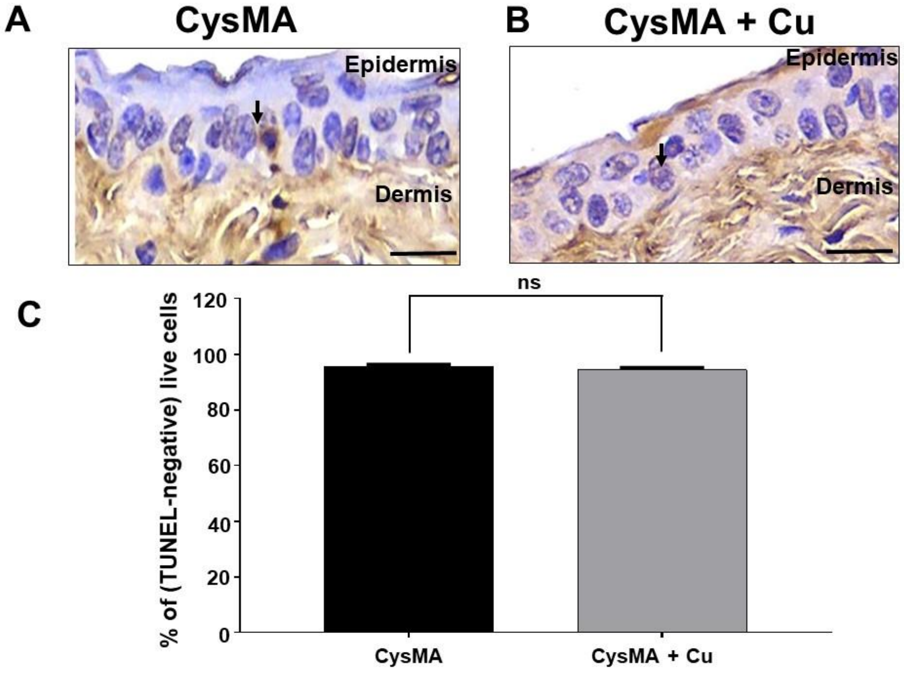

Figure S2. No significant cytotoxicities of hydrogels. (A) Skin histology (TUNEL staining) of mice 24 h after topical application of a hydrogel with (CysMA + Cu) or without (CysMA) cooper. Nuclei of live cells was stained with hematoxylin (blue stains). Arrows indicate the dead cells detected by diaminobenzidine (brown stains). Bars = 30 μm. (B) The percentages of (TUNEL-negative) live cells in skin applied with a hydrogel with or without cooper were quantified. Data are the mean ± SD of three independent experiments. ns = not significant.

References

1. Traisaeng, S.; Herr, D.R.; Kao, H.-J.; Chuang, T.-H.; Huang, C.-M. A Derivative of Butyric Acid, the Fermentation Metabolite of Staphylococcus epidermidis, Inhibits the Growth of a Staphylococcus aureus Strain Isolated from Atopic Dermatitis Patients. *Toxins* **2019**, *11*, 311, doi:10.3390/toxins11060311.
